# Anesthetic management in a spinal cord-injured parturient woman with a left hip resection and secondary scoliosis: A case report

**DOI:** 10.1097/MD.0000000000014527

**Published:** 2019-02-22

**Authors:** Hui Liu, Xuemei Lin, Min Diao, Yushan Ma

**Affiliations:** Department of Anesthesiology, West China Second Hospital of Sichuan University, Key Laboratory of Birth Deficits and Related Diseases of Women and Children, Sichuan University, Ministry of Education, Chengdu, China.

**Keywords:** anesthesia, cesarean delivery, left hip resection, scoliosis, spinal cord-injured

## Abstract

**Rationale:**

Pregnancy after spinal cord injury, hip resection, leg amputation, and scoliosis is an uncommon event. Given the specific pathophysiological changes in this patient, an aesthetic management presented a particular challenge. The effects on the physiological changes associated with pregnancy, aesthetic methods, blood loss, autotransfusion from uterine contractions and thrombotic risk had to be considered.

**Patient concerns:**

A 25-year-old female earthquake survivor was admitted at 36.4 weeks of pregnancy for preterm labor. She had suffered from a spinal cord injury and complex trauma and had subsequently undergone left hip resection, bilateral amputations, and multiple surgical procedures during the previous 6 years. Additionally, she had developed severe scoliosis due to her weight-bearing posture.

**Diagnoses:**

High amputation after earthquake injury; Scoliosis; Vulvar reconstruction; Intrauterine pregnancy (35.6 weeks) with a single live fetus with possible premature delivery.

**Interventions:**

We administered general anesthesia during a cesarean section for the parturient woman. Both the central venous pressure and pleth variability index were used to continuously evaluate intraoperative fluid management and blood loss.

**Outcomes:**

Delivery and patient recovery were uneventful.

**Lessons:**

Anesthetic management of a pregnant woman with a spinal injury, scoliosis, left total leg and right below-knee amputations, and left hip resection requires considerable attention. Advances in medical technology have provided clinicians with insights into managing patients with this condition.

## 1 Introduction

The incidence of spinal cord injury in pregnant women is quite low, and few cases have been reported in the literature.^[1–3]^ In addition, pregnancy after spinal cord injury, hip resection, leg amputation, and secondary scoliosis is an uncommon event. Traumatic lower limb amputees have increased morbidity and mortality due to cardiovascular disease.^[4,5]^ Factors such as insulin resistance, psychological stress, and patient behaviors, may have systemic consequences for the arterial system and may contribute to the increased cardiovascular morbidity in amputees.^[6]^ The devastating 2008 Sichuan Earthquake took over 70,000 lives.^[7]^ We report a cesarean delivery performed in an earthquake survivor who suffered from spinal cord injury and abdominal and bilateral lower extremity trauma and subsequently underwent an exploratory laparotomy, bilateral amputations, left hip resection, and multiple surgical procedures within the previous 6 years. The Ethics Committee of the West China Second Hospital of Sichuan University waived the need for ethical approval for this case report. Informed consent was obtained from the patient.

## 2 Case presentation

A 25-year-old gravida 1 para 0 woman at 35.6 weeks of pregnancy who weighed 57 kilograms was admitted to our hospital because of irregular uterine contractions. The patient had been buried for 48 hours in the ruins after an earthquake. She had undergone more than 30 surgical procedures within the previous 6 years. Her legs and abdomen were severely injured in the earthquake. She underwent left, total leg and right below-knee amputations, left hip resection due to clostridial necrotizing fasciitis, transverse colostomy, cystic and vulvar reconstructions, and colostomy reversal surgery approximately 2 years before becoming pregnant. In addition, she also suffered from spinal shock after the accident. Her sensory and motor function below the level of T10 did not recover until 8 months later. In addition, the patient had progressed to a chronic stage in which her reflex activity was regained. This stage was characterized by disuse atrophy, flexor spasms, and exaggerated reflexes.^[8]^ To bear the weight of her body, she had to sit, lie down, or lie on her side. As a result, the uneven distribution of the stress caused compensatory changes in her spine, leading to severe scoliosis.

**Figure 1:**
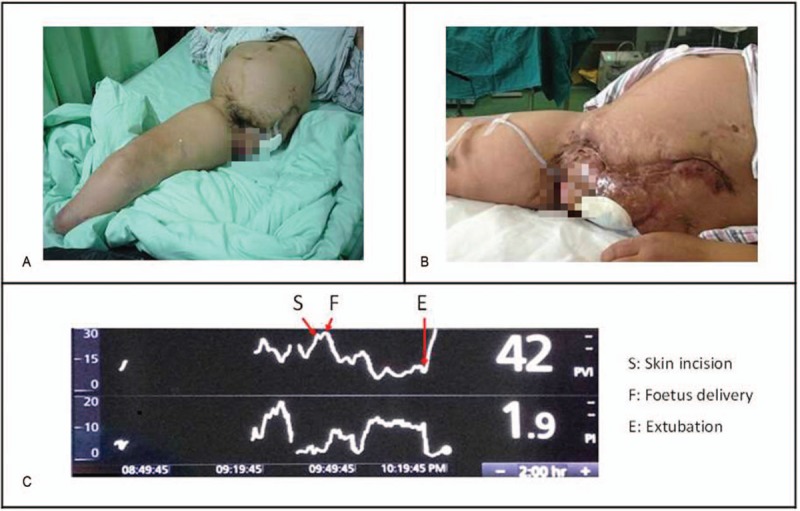
Anesthetic management during a cesarean delivery in a lower limb amputee presented a particular challenge. Panel A, The patient underwent left total leg and right below-knee amputations, left hip resection due to clostridial necrotizing fasciitis, transverse colostomy, cystic and vulvar reconstructions, and colostomy reversal surgery within the previous 6 yr. Panel B, The incomplete pelvic structure due to the previous left hip excision and extensive keloid tissue over the perineal region precluded the possibility of a trial of labor. Panel C, The CVP and PVI were used to guide fluid management intraoperatively. After the delivery of the fetus, the PVI decreased significantly from 2628% to 6–16%. CVP = central venous pressure, PVI = pleth variability index.

This patient had 2 episodes of congestive heart failure during her previous surgical interventions that required hospitalization in an intensive care unit (ICU). When she was admitted to our hospital, the fetal heart rate and movement were monitored closely. Dexamethasone was used to promote fetal lung maturation. At 36.4 weeks of gestation, her uterine contractions became more frequent, and preterm delivery was inevitable. Spontaneous vaginal delivery was not an option because of the patient's incomplete pelvis structure due to the previous left hip resection and extensive keloid tissue over the perineal region (Fig. 1). A cesarean delivery was planned after multidisciplinary consultation and meetings with the patient.

General anesthesia was planned due to the patient's traumatic stress experience. Preoperatively, we ruled out deep venous thrombosis using Doppler sonography. The patient's right internal jugular vein was cannulated preoperatively due to difficult intravenous access. The patient inhaled oxygen in the operating room in the semidecubitus position. In preparation for a potentially prolonged operation, arterial access was also established according to the standard American Society of Anesthesiology monitoring guidelines. We used the central venous pressure (CVP) and pleth variability index (PVI) to guide fluid management. The bispectral index (BIS) and continuous total hemoglobin were also measured. The patient's blood pressure was 105/63 mm Hg, her heart rate was 102 beats/min, her oxygen saturation was 99% when breathing oxygen (>4 L/min), and her CVP was 6 cm H_2_O. After intravenous hydration with 300 ml of lactated Ringer solution (approximately 10 ml/min), she was positioned in the supine position with left uterine displacement to minimize aortocaval compression.

After sterile preparation, general anesthesia was induced through rapid sequence induction with ketamine, propofol, and succinylcholine, following adequate preoxygenation. A surgical incision was made immediately after successful tracheal intubation. To deliver the fetus as quickly as possible, a high transverse abdominal incision and a low transverse segmental uterine incision were used by the obstetric team to avoid surgical scars and peritoneal adhesions. A male infant weighing 2940 g was delivered in 3 minutes. Apgar scores were estimated to be 10 at 1 minute and 10 at 5 minutes. Anesthesia was maintained with a reduced sevoflurane concentration and sufentanil. Warm blankets were used during surgery, and parts of the patient's limbs were protected. The patient was stable during surgery with no significant blood loss. The patient's intraoperative systolic blood pressure was 110 to 130 mm Hg, her heart rate was 100 to 110 beats/min, her BIS was 45 to 65, and her CVP was 5 to 8 cm H_2_O. The duration of surgery and anesthesia were 34 minutes and 1 hour and 42 minutes, respectively. Intraoperatively, the estimated blood loss was approximately 500 ml, 800 ml of lactated Ringer solution was administered during surgery, and 200 ml of clear urine was collected. The patient was extubated after surgery and was then transferred to the ICU. At 42-days of follow up, she exhibited no signs of any postoperative complications.

## 3 Discussion

Current literature was reviewed regarding anesthesia for a cesarean section pregnancy brought to near term in a spinal cord-injured mother with a previous left hip resection and secondary scoliosis. Cross et al^[9]^ reported that approximately 3000 women of childbearing age suffer spinal cord injuries each year in the United States; however, there are few reports in the literature that address pregnancy, labor, and delivery in this patient population. Ehrenberg et al^[10]^ reported the details of a unique case of pregnancy in a spinal cord-injured bilateral total leg amputee. According to their report, the pelvic anatomy of the patient was intact, which was different from our patient's left hip excision and secondary scoliosis. Considering the pathophysiological changes in the patient after multiple surgical procedures, perioperative management in this patient required more considerations and presented a particular challenge.

Traumatic lower limb amputees have increased morbidity and mortality due to cardiovascular disease.^[4,11]^ Several factors including insulin resistance and psychological stress may have systemic consequences in the arterial system and may contribute to increased cardiovascular morbidity in amputees.^[6]^ Significant hypertension was observed in above-knee amputees.^[12]^ Fortunately, our patient's blood pressure remained normal during pregnancy, and no cardiovascular disease was identified before delivery. However, decreases in sympathetic tension below the level of spinal cord injury in pregnant women make these patients prone to postural hypotension, which leads to decreased uterine placental perfusion.^[8]^

The physiologic changes that occur during pregnancy include an increase in minute ventilation and oxygen demand and a decrease in the functional residual capacity (FRC) and the closing capacity of the lungs. The size of the thoracic cage normally increases during pregnancy. If the thoracic cage is relatively fixed by scoliosis, the diaphragm is responsible for all incremental increases in minute ventilation.^[13]^ As pregnancy progresses, the enlarging uterus causes elevation of the diaphragm, further decreasing FRC and closing capacity, which may lead to greater ventilation-perfusion mismatch and decreased arterial oxygen content. Peak increases in pulmonary activity are reached by the middle of the third trimester. Nevertheless, because the uterus continues to grow, it may further encroach on the thorax and cause deterioration, even though the patient's respiratory demand has stabilized.^[13]^ The loss of both FRC and expiratory reserve volume during pregnancy may increase the likelihood of respiratory compromise associated with spinal cord injuries. Our patient's respiratory reserve function was unknown because no lung function examination was performed, and sedative drugs were not administered before surgery. Observing the oxygen saturation after inhaling oxygen and postoperative respiratory conditions in patients have shown that patients with a severe spinal curve but a good cardiopulmonary function can tolerate pregnancy well.^[14]^

Crosby et al^[15]^ reported that pregnancy may aggravate many of the medical complications associated with spinal cord injury. Ghidini et al^[16]^ reported that preterm delivery occurred in 33% of women with spinal cord injuries; 22% were unable to feel preterm labor. Urinary complications (59%), dysreflexia (27%), worsened spasticity (22%), and thrombosis (8%) were the most common complications during pregnancy. Spinal cord injury put our patient at risk of premature birth. Women with spinal cord lesions above T11 have an increased risk of preterm labor because these women do not have intact sensations of uterine activity and labor pain.^[17,18]^ Patients with complete spinal cord injury above T5 have no pain sensation.^[19]^ Our patient's spinal cord injury at the T10 segment allowed her to perceive some uterine contractions, but the pain associated with each uterine contraction was not serious. However, during the chronic stage of spinal cord injury, uterine contractions still lead to autonomic hyperreflexia, and secondary vasoconstriction can lead to fetal hypoxia and bradycardia. Most autonomic hyperreflexia occurs during the period of childbirth.^[20]^ In addition, uterine contractions lead to increased abdominal pressure, which can increase the pulmonary circulation pressure and aggravate the burden of the heart and lungs.

Administration of regional anesthesia is the most common method for the prevention or treatment of autonomic hyperreflexia during labor and delivery. Although the patient had secondary thoracolumbar scoliosis after amputation, this was not a contraindication for regional anesthesia. We considered neuraxial anesthesia initially since blood pressure does not drop significantly after spinal anesthesia in lower limb amputees because of the absence of lower limb vascular dilation. In addition, neuraxial anesthesia is a useful modality for postoperative pain control. However, due to the patient's multiple traumatic experiences, general anesthesia for the relief of maternal discomfort was required when she was admitted to our hospital. After 6 years of living with a spinal cord injury, the patient experienced denervation injury. The depolarizing muscle relaxant succinylcholine was used to facilitate laryngoscopy and intubation. Our results suggested that succinylcholine was safe to use during this period.

The blood volume of patients with spinal cord injury is reduced by approximately 20%.^[19]^ We expected considerable adhesions upon entering the abdomen, and significant blood loss was anticipated. Moreover, due to the patient's previous limb amputations, her total blood volume was estimated to be significantly less than that of a patient with a complete frame. No definitive data exist on the effects of reduced blood volume due to left lower limb and right leg amputations, which accounts for a proportion of blood volume in the body. Hugh et al reported that moderate blood loss, which is expected during delivery, has significant potential to cause cardiovascular instability.^[10]^ To evaluate intraoperative bleeding in real time, hemoglobin was monitored noninvasively and continuously with a hemoglobin monitoring device (Masimo-Rainbow SET pulse oximeters-Radical-7).

Autotransfusion from uterine contractions also needs to be considered. A healthy parturient patient can compensate for the increased burden on the heart during cesarean delivery through lower limb vascular dilation; however, amputee parturient patients might not bear changes in capacity easily and are likely to experience heart failure. In fact, this patient had experienced congestive heart failure twice during her previous surgical interventions and ICU hospitalizations, which had resulted from excessive volume load due to excessive infusions. Therefore, capacity management during this cesarean section was more prudent than in the patient's previous surgeries. CVP and the PVI were used to guide fluid management. It has been shown that the sensitivity and specificity of the PVI for predicting fluid responsiveness are higher than those of CVP, pulmonary capillary wedge pressure, and the cardiac index.^[21]^ In our patient, the PVI decreased significantly from 2628% to 6–16% after delivery of the fetus, while CVP was maintained between 5 and 8 cm H_2_O. The change in the PVI could be explained by the significant autotransfusion (Fig. 1).

Pregnancy in patients with spinal cord injury increases the risk of deep vein thrombosis. Amputees also manifest altered blood coagulability indices, including higher fibrinopeptide A, higher factor VII and lower antithrombin III levels than in healthy subjects.^[4]^ Considering the hypercoagulable state that occurs during pregnancy, thrombosis risk should be considered. Therefore, we ruled out deep venous thrombosis using Doppler sonography preoperatively.

## 4 Conclusion

We demonstrated successful general anesthesia during a cesarean section for a spinal cord-injured parturient patient with a left hip resection and secondary scoliosis. When the patient's condition is stable before labor, a cesarean section can be performed near term, and the outcome of the fetus after general anesthesia is good. The perioperative conditions of these types of patients may not be as poor as expected. Sufficient considerations and preparations must be made before surgery. The patient's vital signs must be closely monitored and controlled and the anesthesia depth ensured, and cooperation between obstetricians and physicians from other related departments is necessary. Advances in medical technology have provided clinicians with more insights for managing this condition.

## Acknowledgments

The authors would like to thank their colleagues working at that time in West China Second Hospital of Sichuan University who assisted in the anesthesia management of this patient.

## Author contributions

**Data curation:** Xuemei Lin, Min Diao.

**Investigation:** Hui Liu.

**Software:** Hui Liu, Min Diao.

**Writing – original draft:** Hui Liu, Yushan Ma.

**Writing – review and editing:** Hui Liu, Xuemei Lin, Yushan Ma.
